# The Dual Therapeutic Potential of Ottelione A on Carbon Tetrachloride-induced Hepatic Toxicity in Mice

**DOI:** 10.1007/s12010-023-04346-8

**Published:** 2023-02-02

**Authors:** Rasha Fekry Zahran, Lina Mahmoud EL-sayed, Thomas Robert Hoye, Seif-Eldin Nasr Ayyad

**Affiliations:** 1 Biochemistry division, Chemistry Department, Faculty of Science, Damietta University, 34517 Damietta, New-Damietta, Egypt; 2 Departments of Chemistry and Medicinal Chemistry, University of Minnesota, 55455 Minneapolis, MN USA; 3 Department of Chemistry, Faculty of Science, Damietta University, New Damietta, Egypt

**Keywords:** Hepatoxicity, CCl_4_, *Ottelia allismoide*, TGFß1, Hepato-protective, Oxidative stress

## Abstract

**Background:**

Some herbal natural products play an important role in protecting organisms from the toxic effect of some xenobiotics. The present study was designed to evaluate the potential therapeutic effects of Ottelione A (OTTE) against carbon tetrachloride(CCl_4_)-induced toxicity in mice.

**Methods:**

Adult male Swiss albino mice were divided into six groups: group I was used as a normal control received olive oil; group II received DMSO; group III received OTTE; group IV received CCl_4_ in olive oil, (injected i.p) 3 times/week for 6 weeks; group V received the same CCl_4_ regimen as group IV followed by OTTE injected for 15 days, and group VI first received OTTE injected for 15 days followed by the same CCl_4_ regimen as group IV. Some biochemical and histological parameters were investigated.

**Results:**

Our results showed that the administration of CCl_4_ caused hepatotoxicity, as monitored by the significant increase in biochemical parameters concerning the olive oil group. Treatment with OTTE appeare d to be effective against hepatotoxic and liver changes induced by CCl_4_, as evidenced by the improvement of the same parameters.

**Conclusion:**

Ottelione A (OTTE) has good antioxidant and therapeutic properties, which can help in preventing CCl_4_-induced hepatotoxicity in both pre-treatment and post-treatment modes.

**Graphical Abstract:**

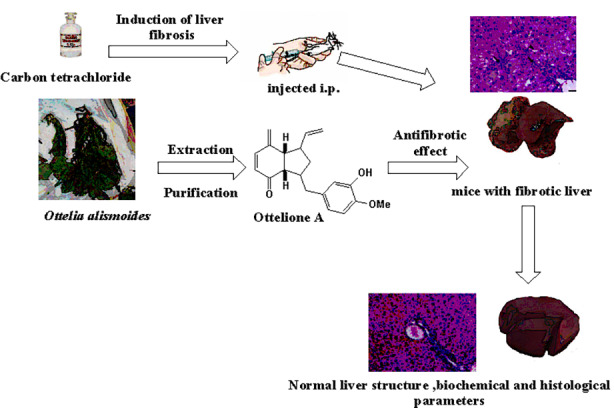

## Introduction

Liver is an essential organ responsible for a variety of critical biochemical and physiological phenomena, such as the metabolism and detoxification of endogenous (waste metabolites) and exogenous (toxic) substances, such as medications, xenobiotics, and homeostasis [1]. Since liver cells are the primary sites for the metabolism of exogenous chemicals, they are often exposed to high concentrations of these chemicals, resulting in liver failure, liver cell damage, or hepatotoxicity [2]. A major cause of death around the world is hepatotoxicity [3].

Fibrosis in mice can be induced by using Carbon tetrachloride (CCl_4_) [4]. The toxicity of CCl_4_ is caused by the generation of free radicals and alterations in the antioxidant system of tissues [5]. Cytochrome P450 bio-activates CCl_4_ in the body to form the carbon trichloride radical and chlorine. Lipid peroxidation causes significant cellular damage by altering membrane characteristics. [6]. trichloromethyl-peroxyl the more active derivative of carbon trichloride radical may also covalently bond to lipids and microsomal proteins, causing the production of reactive oxygen species such as superoxide and hydroxyl radicals, which attack liver macromolecules [7].

New medications to treat liver disorders must be sought to improve the commonly used medicines, which are of dubious effectiveness and protection [8]. Dietary natural products with antioxidant properties are known to have therapeutic value [9]. These natural products have been shown to protect against a variety of diseases [10]. The medicinal properties of the plants have also been studied due to their potentially potent pharmacological activity, low toxicity, and/or economic feasibility compared to synthetic drugs [11].

*Ottelia alismoides* is a partially flooded water plant .it found in Egypt every summer in rice fields and irrigation ditches [12]. This plant includes natural chemicals as flavonoids, terpenoids, tannins, glycosides, alkaloids, and phenolic compounds [13]. *Ottelia alismoides* contains two diastereomeric *otteliones* A (OTTE), and B. An extract of *Ottelia alismoides* yielded ten novel diarylheptanoids [14].

The entire plant has been used as a drug in the following applications: its anti-tuberculosis activity [15]; ability to heal abscesses of breast cancer; ulcers; burns; treatment of asthma; topical application for skin diseases and hemorrhoids; as a poultice against fever [16]; and as a treatment for diabetes [17]. More specifically, OTTE itself has a powerful tubulin polymerization inhibitory effect and cytotoxicity in tumor cell lines in vitro [18, 19].

Owing to the significant latent of OTTE as a medicinal agent, this study was designed to elucidate details of bioactivity of OTTE by studying its effects as either a pre-or post-treatment on different oxidative stress, inflammatory, fibrosis, and hepatic markers in an experimental animal model of CCl_4_-induced liver toxicity.

## Materials and Methods

### Reagents

All chemicals were of high purity as CCl_4_, 99+%, extra pure was obtained from BDH Chemicals, Ltd., Poole, England. CCl_4_ was diluted 1:3 with virgin olive oil. Boric acid powder and vanillin were obtained from El-Gomhouria Company for Drugs and Chemicals, Egypt. Dimethyl sulfoxide (DMSO) ≥ 99.5% (GC), diethyl ether ≥ 99.9%, acetone ≥ 99.5% (GC), ethanol ≥ 99.8% (GC) and ethyl acetate ≥ 99.5% (GC) were purchased from Sigma-Aldrich Chemical Co., (St Louis, MO, USA). Acetylacetone (2, 4-pentanedione), 99+% was obtained from ACROS ORGANICS, (Geel, Belgium). Hydrochloric acid, 37%, extra pure, petroleum ether, 99+%, extra pure, methanol, 99.8%, extra pure and sodium sulfate, anhydrous, 99+%, extra pure were purchased from Fisher Scientific UK. Kits used for the experiments of biochemical studies were purchased from Biodiagnostic Company, Dokki, Giza, Egypt and Bioassay Technology Laboratory CO., Shanghai, China.

### Preparation of Plant Extract

Whole plants of *Ottelia alismoides* (350 g) were collected from canals of the River Nile, Delta, Egypt during the summer season. Plants were air-dried at room temperature and stored at -20 ˚C until extracted. An initial *Ottelia alismoides* extract, obtained by exhaustive extraction with petroleum ether, was subjective to consecutive column chromatographies on silica gel, before final preparative thin-layer chromatography (TLC) to provide pure OTTE. The identity of OTTE was confirmed by proton nuclear magnetic resonance ( ^1^ H NMR) analysis according to Ayyad et al. [18]. The pure Ottelione was dissolved in DMSO (1%) and diluted to the necessary levels [12].

### Ethics approval and consent to participate

All animal experiments conformed to the British Home Office Regulations (Animal Scientific Procedures Act 1986) and associated guidelines, EU Directive 2010/63/EU for animal experiments. This work launched after attaining permission from the scientific and ethical committees of Faculty of Science.

### Experimental Animals

The study design used 66 adult male Swiss albino mice weighing 22–25 g. These were supplied by the Zoology Department, Faculty of Science, Egypt. The animals were housed in wire-mesh cages (11 mice per cage) in the Animal House of the Faculty of Science and administered food and filtered water. They were adapted for a week before the beginning of the experiment.

### Experimental Design

The effects of CCl_4_ and OTTE on oxidative stress, hepatic injury, liver enzymes, and antioxidant activity of male mice were studied by dividing the animals into six groups of eleven animals each.

- Group I: Vehicle control group I (olive oil group) in which mice received an intraperitoneal injection (i.p.) of 0.025 mL of olive oil (vehicle #1) three times per week for six weeks.
- Group II: Vehicle control group II (DMSO group) in which mice received daily i.p. (1%) DMSO (vehicle #2, 0.3 mL) for 15 days.
- Group III: Ottelione A only group (OTTE group) in which mice received daily i.p. OTTE in DMSO (0.3 mL) for 15 days.
- Group IV: Positive control group (CCl_4_ group) in which mice received i.p. 0.025 mL 1:3 v/v of CCl_4_ in olive oil three times per week for six weeks.
- Group V: Post-treatment group in which mice received i.p. (0.025 mL) 1:3 v/v of CCl_4_ in olive oil three times per week for six weeks, followed by daily i.p. OTTE in DMSO (0.3 mL) for 15 days.
- Group VI: Pre-treatment group in which mice received daily i.p. OTTE in DMSO (0.3 mL) for 15 days followed by i.p. (0.025 mL) 1:3 v/v of CCl_4_ in olive oil three times per week for six weeks.

At the end of the experimental phase, the whole blood was collected by heart penetration for biochemical examination. a portion of the liver was stored at − 20 °C until the antioxidant parameters were measured, and the second portion of the liver tissue was attached in (10%) formalin for histopathological evaluation.

### Liver Function Enzymes

The activities of serum alanine transaminase (ALT) and aspartate transaminase (AST) were estimated by using Biodiagnostic kit, Egypt (CAT.NO. AT1034), according to the method of Reitman and Frankel [20]. Serum total protein and albumin levels were estimated using kits from Biodiagnostic, Giza, Egypt (CAT.NO. TP2020, CAT.NO. AB1010), according to the method of Gornall et al. [21] and Doumas et al. [22].

### Liver Total Protein (TP) Estimation

Total protein in tissue homogenate content was estimated according to the procedure of Lowry et al. [23].

### Nucleic Acids in Liver Tissue

The extraction and quantification of nucleic acid were done using the method of Price et al. [24]. The DNA content of the extract was colorimetrically determined using the diphenylamine according to a method of Zhao et al. [25], and the RNA content was estimated by using orcinol according to the method of Benthin et al. [26].

### Extraction and Quantification of Total Lipids in Liver Tissues

Total lipids in liver tissues were evaluated using the method of Izard and Limberger [27].

### Immunoassay of Transforming Growth Factor - ß (TGF-ß1) Levels

TGF-ß1 in tissue homogenate of the liver was assayed by an ELISA technique according to the manufacturer’s protocol of Bioassay Technology Laboratory CO., Shanghai, China (CAT.NO. E0660Mo).

### Assessment of Oxidant/Antioxidant Status Biomarkers

Liver reduced glutathione (GSH) concentration in tissue homogenate was estimated by using a commercial kit from Biodiagnostic, Company, Dokki, Giza, Egypt (CAT. NO. GR2511), according to the method of Beutler and Kelly [28]. Plasma total antioxidant capacity (TAC), and superoxide dismutase (SOD) activity were colorimetrically assayed using commercial kits supplied by Biodiagnostic, Giza, Egypt (CAT. NO. TA2513, CAT. NO. SD2521), according to the method of koracevic et al. [29] and Dechatelet et al. [30].

The assay of catalase (CAT) activity in tissue homogenate of the liver was obtained by the method described by Aebi [31]. Lipid peroxidation or MDA activity was determined by the measurement of thiobarbituric acid reactivity. MDA in RBCs was determined using the method described by Ohkawa et al. [32]. Assay of nitric oxide (NO) in tissue homogenate of the liver was done according to the method of Montgomery and Dymock [33].

### Histopathological Studies

A sample of liver obtained after decapitation was washed in saline and fixed immediately in 10% formalin for the hematoxylin and eosin (H&E) staining and histopathological analysis. Ascending series of alcohol was used for dehydration of fixed liver tissue samples, which were then cleaned with xylene and embedded in paraffin wax. A rotary microtone was used to prepare 5–6 μm thickness sections of the tissues and stained with H&E dye according to standard protocols of Oner-lyidogan et al. [34].

### Statistical Analysis

All the data are expressed as means ± SD and the statistical analysis was done by SPSS® Statistics, version 22. One-way analysis (ANOVA) was performed for testing the significance of the treatment. Differences among the group means were determined by Tukey’s test. Values of *P* were considered significant at levels ≤ 0.05.

## Results

### Effects of Treatments on Liver Function

Table 1 showed that there was no significant change in ALT and AST between OTTE and the olive oil group (*p* = 0.6 and *p* = 0.4, respectively). Administration of CCl_4_ resulted a significant increase in hepatic enzyme activities (ALT and AST) when compared to the normal mice (*p* = 0.0001). On the other hand, either post- or pre-treating mice with OTTE exhibited significant improvement in hepatic enzymes compared to CCl_4_ (*p* = 0.0001) alone. Post-treatment with OTTE has an insignificant elevation in ALT and AST compared to normal (*p* = 0.9 and *p* = 0.8, respectively).

**Table 1:** Effect of CCl_4_ and OTTE treatment on the biochemical and hepatotoxicity parameters against hepatotoxicity in albino mice

	Olive oil GI	DMSO GII	OTTE GIII	CCl_4_ GIV	Post-OTTE GV	Pre-OTTE GVI
**ALT** (U/L)	49.8 ± 3.9	43.7 ± 5.2^a^	47.1 ± 6.2	80.2 ± 2.6 ^a^	50.8 ± 4 ^b^	57.3 ± 1.36 ^a,b^
**AST** (U/L)	55 **±** 1.97	52 **±** 1.41^a^	53 **±** 1.61	89 ± 4.14 ^a^	56 ± 1.4 ^b^	60 ± 1.29 ^a,b^
**Albumin** (g/dl)	4.07 ± 0.68	3.25 ± 0.18^a^	3.7 ± 0.48	1.9 ± 0.12 ^a^	3.79 ± 0.29 ^b^	2.96 ± 0.24 ^a,b^
**Total protein** (g/dl)	6.57 ± 0.55	5.66 ± 0.33^a^	5.78 ± 0.43^a^	3.87 ± 0.35 ^a^	6.19 ± 0.38 ^b^	5.69 ± 0.19 ^a,b^
**DNA** (µg/tissue)	100.8 ± 3.9	110.6 ± 3.1^a^	108.4 ± 2.7^a^	201.7 ± 12.4^a^	113.0 ± 2.08^a,b^	120.5 ± 3.2^a,b^
**RNA** (µg/tissue)	127.3 ± 8.1	132.7 ± 7.3	136.8 ± 4.8^a^	236.8 ± 8.7 ^a^	141.8 ± 2.1^a,b^	146.5 ± 5.1^a,b^
**Total Lipid** (mg%)	80.9 ± 2.3	83.7 ± 0.64	81.8 ± 0.82	224.9 ± 13.1^a^	86.2 ± 1.36 ^b^	96.0 ± 6.8^a,b^
**Total protein** (mg% tissue)	7.85 ± 0.41	7.53 ± 1.2	8.08 ± 0.31	5.13 ± 0.46 ^a^	7.89 ± 0.23 ^b^	6.54 ± 0.58 ^a,b^

Mean ± standard deviation followed by different letters in the column differs from each other by Tukey’s test, a = significant when compared with the olive oil group, b = significant when compared with the CCl_4_ treated mice GIV. a, GII versus GI, GIII versus GI, GIV versus GI, GV versus GI, GVI versus GI; b, GV versus GIV, GVI versus GIV; p > 0.05 is considered non-significant; p < 0.05 is considered significant; p < 0.001 is considered ‎extremely significant. ‎ ALT = alanine amino transaminase; AST = aspartate amino transaminase; DNA = deoxyribonucleic acid; RNA = Ribonucleic acid; GI = Olive oil group; GII = DMSO group; GIII = OTTE group; GIV = CCl_4_ group; GV = Post-treatment group; GVI = Pre-treatment group

Treating mice with CCl_4_ alone decreased both total protein and albumin compared to olive oil group mice (*p* = 0.0001). However, administration of OTTE after CCl_4_ injection caused insignificant changes in serum levels of total protein and albumin as compared to the olive oil group (*p* = 0.5 and *p* = 0.2, respectively). All OTTE-treated groups have a significant elevation in serum protein and albumin compared to the CCl_4_ positive control group.

### Effect of Treatment on Hepatic Nucleic Acids, Total Lipids, and Protein Content

DNA, RNA, and total lipid with a concurrent decrease in the total protein contents in the liver homogenate were observed in the CCl_4_ group relative to the olive oil group (*p* = 0.0001) as shown in Table 1. However, administration of the OTTE (post- and pre-treatment) with CCl_4_ significantly (*p* = 0.0001) decreased DNA, RNA, and total lipid content in the liver as compared to CCl_4_ treated mice. Total protein tissue content significantly increased in all OTTE-treated mice compared to the positive control group (*p* = 0.0001). An insignificant increase in both total lipids and protein content was observed in post-treated mice with OTTE compared to olive oil group (*p* = 0.3, and *p* = 1, respectively, Table 1).

### Effects of Treatments on Hepatic Oxidative Stress Parameters

The data shown in Table 2 indicate that giving CCl_4_ initiated a significant elevation in the hepatic MDA and NO levels as lipid peroxidation index (*p* = 0.0001), down-regulated TAC, SOD, CAT, and GSH activity (*p* = 0.0001) when compared to that of the olive oil group. Whereas post- or pre-treatment of OTTE followed by CCl_4_ produced a significant improvement in the hepatic antioxidant protection mechanism by increasing the hepatic TAC, GSH, SOD, and CAT activities (*p* = 0.0001), at the same time, they induced a significant reduction in the hepatic MDA and NO level as regard to those of the CCl_4_ group (*p* = 0.0001). Furthermore, treatment of OTTE in the post-treatment group was more effective than that of the pre-treatment group.

**Table 2:** Effect of CCl_4_ and OTTE treatment on the oxidative and fibrosis markers against hepatotoxicity of the liver

	Olive oil GI	DMSO GII	OTTE GIII	CCl_4_ GIV	Post OTTE GV	Pre OTTE GVI
**TAC** (Mm/L)	1.63 ± 0.05	1.53 ± 0.03 ^a^	1.48 ± 0.07^a^	0.66 ± 0.05 ^a^	1.33 ± 0.06 ^a,b^	1.03 ± 0.07 ^a,b^
**SOD** (%inhibition)	95.9 ± 0.43	88.1 ± 0.76 ^a^	87.7 ± 1.4 ^a^	60.5 ± 1.2 ^a^	930.0 ± 0.88 ^a,b^	89.6 ± 0.99 ^a,b^
**GSH** (mg/g tissue)	9.45 ± 0.92	7.3 ± 0.67 ^a^	7.6 ± 0.65 ^a^	4.26 ± 0.3 ^a^	8.48 ± 0.34 ^a,b^	7.42 ± 0.54 ^a,b^
**CAT**(U/g tissue)	20.07 ± 0.17	1.93 ± 0.01 ^a^	1.96 ± 0.02^a^	10.07 ± 0.05^a^	1.97 ± 0.01^a,b^	1.94 ± 0.03 ^a,b^
**NO** (µmol/L)	19.6 ± 1.1	21.8 ± 0.5^a^	22.5 ± 0.72^a^	45.4 ± 2.0 ^a^	240.0 ± 0.58 ^a,b^	30.5 ± 1.22^a,b^
**MDA**(umol/ml packed cell)	8.5 ± 1.14	6.9 ± 0.63 ^a^	7.47 ± 0.69	20.1 ± 1.27 ^a^	8.71 ± 0.78 ^b^	11.5 ± 1.08 ^a,b^
**TGFβ1** (pg/g protein)	552.1 ± 71	568 ± 2.5	574 ± 2.3	1627 ± 40 ^a^	563 ± 3.2 ^b^	596 ± 24 ^a,b^

Mean ± standard deviation followed by different letters in the column differs from each other by Tukey’s test, a = significant when compared with the olive oil group, b = significant when compared with the CCl_4_ treated mice GIV. a, GII versus GI, GIII versus GI, GIV versus GI, GV versus GI, GVI versus GI; b, GV versus GIV, GVI versus GIV; p > 0.05 is considered non-significant; p < 0.05 is considered significant; p < 0.001 is considered ‎extremely significant.‎TAC = total antioxidant capacity; SOD = superoxide dismutase; GSH = glutathione reductase; CAT = catalase; NO = nitric oxide; MDA = malondialdehyde; TGF-ß1 = transforming growth factor ; GI **=** olive oil group; GII **=** DMSO group; GIII **=** OTTE group; GIV **=** CCl_4_ group ; GV = post-treatment group; GVI **=** pre-treatment group

### Estimation of Transforming Growth Factor (TGF-ß1)

Data in Table 2 show the influence of OTTE on TGF-ß1 levels in CCl_4_-induced hepatotoxicity. There was an insignificant increase in TGF-ß1 in mice treated with either DMSO or OTTE compared to olive oil group (*p* = 0.1, and *p* = 0.3, respectively). The mean values of TGF-ß1 in liver tissue homogenate in the positive control group (CCl_4_ group) were significantly (*p* = 0.0001) elevated compared to that of the olive oil group. Nonetheless, treating the CCl_4_-induced hepatotoxicity in post-treatment or pretreatment groups with OTTE resulted in a significant (*p* = 0.0001) reduction in TGF-ß1 than that of the positive control group (CCl_4_ group). Post-treatment was more efficient than pre-treatment in ameliorating the levels of TGF-ß1 in liver tissue homogenate, as there was no significant change compared to the normal (*p* = 0.7).

### Effect of Treatment on Histological Analysis

**Figure 1:**
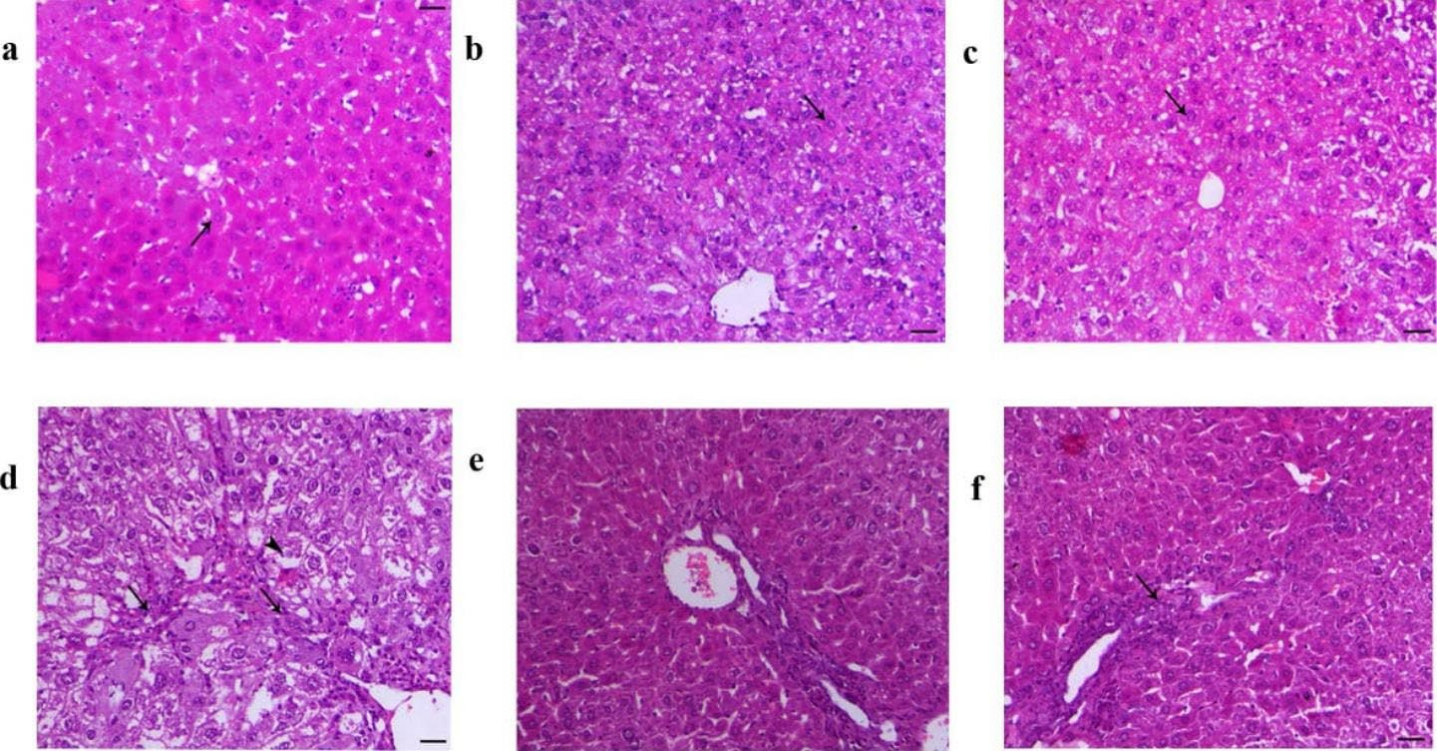
Effect of CCl_4_ and OTTE treatment on Liver histopathology. (a) (GI) olive oil group mice liver showing normal hepatocytes arranged in cords around the central vein (arrow), H&E, X200 bar = 50 μm; (b)(GII) DMSO control mice liver showing normal hepatocytes arranged in cords around the central vein (arrow), H&E, X200 bar = 50 μm; (c) (GIII) Liver of the OTTE group showing normal hepatocytes arranged in cords around the central vein (arrow), H&E, X200 bar = 50 μm; (d) (GIV)Liver of the CCl_4_ group showing extensive hepatic degenerative changes (arrowhead) associated with marked interlobular and intralobular hepatic fibrosis (arrow), H&E, X200 bar = 50 μm; (e) (GV)Liver of post-treatment group showing normal hepatocytes structure H&E, X200 bar = 50 μm; (f)(GVI) Liver of the pre-treatment group showing a marked decrease of hepatic tissue damage (arrow), H&E, X200 bar = 50 μm

The histological examination of liver tissues confirmed the biochemical study in all of the groups as shown in Fig. 1. The histological examination of the Olive oil group, DMSO groups, and OTTE control, showed normal hepatocytes. However, the hepatic injury in the mice treated with CCl_4_ demonstrated extensive hepatic degenerative changes associated with marked interlobular and intralobular hepatic fibrosis. Remarkably, the liver of the post-treatment group showed a marked decrease in fibrosis and a normal liver structure. Liver of the pre-treatment group exhibited a marked decrease in hepatic tissue damage.

**Figure 2:**
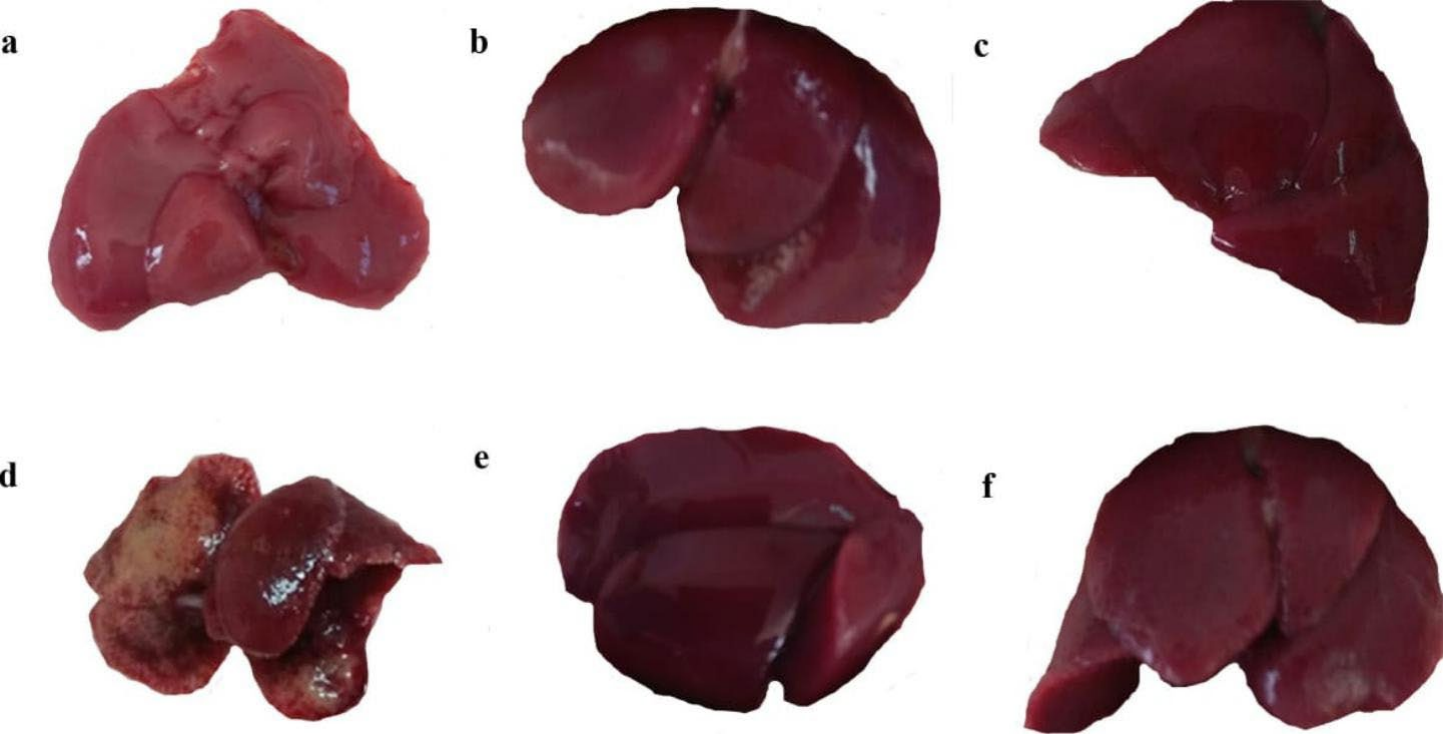
Effect of CCl_4_ and OTTE treatment on the morphological structure of the liver (a) (GI) olive oil group; (b) (GII) DMSO group; (c) (GIII) Liver of OTTE group; (d) (GIV) Liver of CCl_4_ group; (e) (GV)Liver of post-treatment group; (f) (GVI) Liver of pre-treatment group

The livers obtained from the CCl_4_ group were characterized by a flattened, fractured surface, and were dark red, whereas those of the olive oil group exhibited a smooth surface and a bright red color. Both post- and pre-treatments resulted in an improvement in the macroscopic condition of the liver as compared to the CCl_4_-treated mice as shown in Fig. 2.

## Discussion

The metabolism and detoxification of drugs or xenobiotics in the liver can generate toxic free radicals. These free radicals bind to macromolecules and cause oxidative damage to hepatocytes, leading to hepatic injury. Hepatoprotective compounds obtained from plant sources are of great interest due to their negative rather than positive effect inside the body, which is distinct from synthetic drugs [35]. The main goal of this study was to find out if OTTE could protect the livers of mice from the damage caused by CCl_4_ by the production of highly reactive CCl3 and CCl3OO radicals during CCl_4_ metabolism in mice [36].

The degree of hepatic damage is determined by estimating the serum levels of liver biomarkers such as ALT, AST, albumin, and total protein [37]. Liver biomarkers are present in the mitochondria of hepatocytes. However, CCl_4_ damages the hepatocyte membrane, leading to loss of structural integrity and leakage of liver enzymes from the mitochondria into the blood circulation. Significant increases in the levels of liver biomarkers ALT and AST, along with a significant reduction of albumin and total protein, were observed in the group of mice treated with CCl_4_. But after treatment with OTTE, most of these changes went away, which showed that the liver functions were back to normal.

Metabolic activation is one of the major mechanisms for drug-induced hepatotoxicity and has been received more and more attention in recent years [38]. Reactive metabolic intermediates generated play a critical role in drug induced hepatotoxicity by adduction with liver protein [39]. So, CCl_4_ treatment caused a significant reduction in hepatic protein content that correlated with the rapid loss of the ability of the liver to synthesize albumin and total protein, causing an overall significant decrease in the total protein and serum albumin levels. Successive generation of free radicals through the administration of CCl_4_ forms covalent bonds with hepatocyte proteins, causing inhibition of protein synthesis [40]. The treatment of mice with OTTE elevated the total protein content, especially in the post-treated mice, indicating the ability of OTTE to enhance the hepatocyte injury.

The results of the present study have also suggested that CCl_4_ treatment could have affected the lipid metabolism of the liver. It can be assumed that hyperlipidemia in CCl_4_ mice resulted from damage of hepatic parenchymal cells that led to disturbances of lipid metabolism in the liver [41]. Nevertheless, mice treated with OTTE showed a significant decline in total lipid values compared to CCl_4_-treated mice, suggesting lipid-lowering effects of OTTE in the liver. These significant effects might be due to the quenching of free radicals, leading to a decrease in hepatic lipid peroxide. In this regard, OTTE might be considered a natural antioxidant.

 CCl_4_ treatment resulted in significant DNA and RNA elevation compared to olive oil group. One can suggest for the CCl_4_ maybe trigger DNA and RNA damage that leads to the elevation of its fragmentation in hepatic tissue. However, the post-treatment of mice with OTTE reversed the damaging effect of CCl_4_ on hepatocyte’s nucleic acids.

 The CCl_4_ group increased levels of lipid peroxidation (MDA and NO), and decreased the antioxidant parameters SOD, CAT, GSH, and TAC compared to the olive group. These observations are in accordance with the previous study reported by Abo-Zaid et al. [42]. CCl_4_ toxicity causes free radical reactions through enzymes of the CYP 450 system, producing hepatotoxic metabolites. These metabolites cause hepatotoxicity through induction of lipid peroxidation, that all together gives MDA and NO products that serve as an indicator of hepatic toxicity and failure of protective mechanisms to delay the free radical formation [43]. OTTE reduces the MDA and NO in the liver significantly as compared to the CCl_4_-treated group at post-treatment, thus the OTTE has a therapeutic efficacy. This can be explained by the inhibition of lipid peroxidation in the liver. OTTE reduces the MDA and NO in the liver significantly as compared to the CCl_4_-treated group at post-treatment.

There is a need to investigate OTTE’s antioxidant activity, which might limit the production of free radicals against CCl_4_-induced hepatotoxicity. Oxidative stress was destroyed by the body’s defense mechanism, which is done by a collection of antioxidant enzymes. SOD and GSH are the principal enzymes involved in the clearance of hazardous compounds, which are the primary cause of CCl_4_-induced liver disease [44]. It is worth noting that the CCl_4_-treated group initiates lipid peroxidation while also decreasing tissue TAC, CAT, GSH, and SOD activities. These findings may corroborate those of Chao et al., who found that CCl4 therapy causes GSH depletion, CAT with SOD, and liver damage [45].

 The present study had indicated the marked re-establishment of antioxidant enzymes TAC, SOD, CAT, and GSH in hepatic tissue near to their normal value due to post-treatment or pretreated by OTTE. The previous results confirm that OTTE retention of antioxidant constituents at post-treatment has been proposed as an effective therapeutic attitude towards hepatic damages, therefore hepatotoxicity CCl_4_-induced in mice model. Moreover, the improvement of the oxidative enzymes in pretreated mice with OTTE followed by supplementation of CCl_4_ suggested a protective effect on hepatocytes. These results may confirm those of El-Missiry et al.[12] who demonstrated that treatment with OTTE leads to increased levels of GSH, SOD, and CAT as well as decreased lipid peroxidation.

 Hepatic fibrosis is associated with chronic inflammatory disorders, which are defined by the loss of hepatocytes and changes in hepatic structure due to the inequity between extracellular matrix synthesis and scar tissue development [46]. Moreover, TGF-β1 is a vital pro-inflammatory and fibrogenic cytokine in liver fibrosis [47], The data of this study showed a significant increase in tissue TGF-β1 in the positive control (CCl_4_) group. These results agree with those of Hafez et al. [48] who reported that CCl_4_ induction can cause fibrosis of hepatocytes via enhanced production of pro-fibrogenic factors such as TGF-β1 and TNF-α. Furthermore, our study revealed that OTTE significantly reduced the levels of TGF-β1 in the tissue of the positive group, CCl_4_. Those results follow other studies that reported that a natural product inhibits the profibrotic activity of TGF-β1 on renal fibroblasts [49].

 Importantly, the hepatic histology of the normal and OTTE control groups showed normal morphology in the liver tissue. However, the mice exposed to CCl_4_ showed severe hepatic degenerative disease associated with marked interlobular and intralobular hepatic fibrosis, compared to the control groups. The latter pathological changes as a result of CCl_4_ hepatotoxicity agree with Wu et al. [50]. Therefore, the improvement and fibrosis reduction induced upon post-treatment with OTTE could be due to its large antioxidant content. Also, the pretreated OTTE showed a noticeable decrease in hepatic tissue damage and nearly restored them to normal, confirming the protective effect of the OTTE on the liver due to their higher content of antioxidant substances such as flavonoids and phenolic compounds.

## Conclusions

 OTTE mediates several hepatic therapeutic mechanisms in hepatotoxic mice. The animals show improvements in hematological parameters, hepatocyte functionality, anti-hyperlipidemia, anti-inflammatory, anti-fibrotic, and antioxidant mechanisms. Additionally, the results may point to the involvement of OTTE in treating hepatocytes, and this was also confirmed by its effect on regenerating liver cells by histological analysis of liver tissue sections.

## Data Availability

The authors declare that all generated and analyzed data are included in the.

## Abbreviations

ALT: Alanine transaminase
AST: Aspartate transaminase
CAT: Catalase
CCl_3_: Carbon trichloride radical
CCl_3_OO: Trichloromethyl peroxyl
CCl4: Carbon tetrachloride
Cyp4502E1: Cytochrome P4502E1
DMSO: Dimethyl sulfoxide
DNA: Deoxyribonucleic acid
GSH: Reduced glutathione
HE: Hematoxylin-eosin
^1^H NMR: Proton nuclear magnetic resonance
i.p: Intraperitoneal injection
MDA: Malondialdehyde
NO: Nitric oxide
OTTE: Ottelione A
RNA: Ribonucleic acid
ROS: Reactive oxygen species
SOD: Superoxide dismutase
TAC: Total antioxidant capacity
TGF-ß1: Transforming Growth Factor
TLC: Thin-layer chromatography
TNF-α: Tumor necrosis factor alpha
